# Ecomorphology and Morphological Disparity of Caquetaia Kraussii (Perciformes: Cichlidae) in Colombia

**DOI:** 10.3390/ani12233438

**Published:** 2022-12-06

**Authors:** Jordan Hernandez, Amado Villalobos-Leiva, Adriana Bermúdez, Daniela Ahumada-C, Manuel J. Suazo, Margarita Correa, Angie Díaz, Hugo A. Benítez

**Affiliations:** 1 Grupo de Investigación en Biología Descriptiva y Aplicada, Programa de Biología, Universidad de Cartagena, Cartagena de Indias 130015, Colombia; 2 Laboratorio de Ecología y Morfometría Evolutiva, Centro de Investigación de Estudios Avanzados del Maule, Instituto Milenio Biodiversidad de Ecosistemas Antárticos y Subantárticos (BASE), Universidad Católica del Maule, Talca 3466706, Chile; 3 Departamento de Zoología, Facultad de Ciencias Naturales y Oceanográficas, Universidad de Concepción, Concepción 4070386, Chile; 4 Grupo de Investigación Hidrobiología, Universidad de Cartagena, Cartagena de Indias 130015, Colombia; 5 Instituto de Alta Investigación, Universidad de Tarapacá, Casilla 7D, Arica 1000000, Chile; 6 Centro de Investigación en Recursos Naturales y Sustentabilidad (CIRENYS), Universidad Bernardo O’Higgins, Avenida Viel 1497, Santiago 8370993, Chile

**Keywords:** body shape, ecomorphology, geometric morphometrics, Cichlidae, phenotype

## Abstract

**Simple Summary:**

Morphological variation studies allow us to understand the mechanisms and factors that generate variations in populations, thus allowing the development of conservation plans and sustainable management of species. The “yellow mojarra”, Caquetaia kraussii (Perciformes: Cichlidae), is a species native to Colombia and Venezuela that recently has become an essential species in the food security of many Colombian fishing families. Geometric morphometrics (GM) is a tool that quantifies the variation of a shape through multivariate analysis, allowing its graphical and easy visualization. To measure the phenotypic variation of C. kraussii in different localities in “Canal del Dique”, Colombia, advanced morphological tools were used to quantify fish shape and evaluate associations between different ecological parameters of the study sites and the morphological differentiation of their populations. Morphological differences between localities were found and the influence of environmental parameters on the geometric shape of *C. kraussii* was also evidenced. These results demonstrates the efficiency of combined geometrics morphometrics with ecomorphological analysis to evaluate the impact of environmental pressures on fish body shape.

**Abstract:**

Understanding the interspecific morphological variability of Caquetaia kraussii (Perciformes: Cichlidae) between different localities in its distribution range is becoming essential, as this species constitutes a valuable resource for the economy and subsistence of the local human communities where it is endemic in Colombia and Venezuela. In order to develop efficient farming and handling plans for this species, a deep understanding of the factors and mechanisms generating morphological variability is crucial. This study analyzes the morphological variability of *C. kraussii* by using geometric morphometrics in four localities distributed between the Dique and North channels, which are part of the Bolívar department in Colombia. Likewise, the effect of environmental variables such as temperature (T°), dissolved oxygen (OD) and pH on morphological variability was analyzed using a partial least squares approach. The results show that environmental stress has an influence on ~10% of the body shape of *C. kraussii*, whereas ~90% of the body shape is not directly influenced by environmental parameters, suggesting an effect from stress related to sexual dimorphism. Similarly, the analyses show shape variation among localities, mainly between populations of lotic environments and those of lentic environments. This morphological disparity seems to be subject to environmental and sexual stresses in the different localities.

## 1. Introduction

Describing and studying morphology in detail, as well as understanding its origins, has been the base of taxonomic classification and biological diversity studies for centuries [1]. Since morphological variation may be influenced by genetic composition and/or environmental factors and these changes are frequently correlated with ethological, physiological, ecological and life-history traits [2,3,4], understanding morphological changes is a task relevant to determining the dynamics of the populations [5,6]. Carvalho [7] suggested that fish have a great sensitivity to environmental changes and, therefore, have a greater tendency to vary morphologically compared to other vertebrates. Furthermore, given their many living conditions, they can be significantly influenced by a variety of environmental factors (salinity, temperature, oxygen, currents and depth, among others). Thus, morphological variation of fish populations may be an efficient tool to understand evolutionary divergence in different habitats [8,9]. Ecomorphology helps to elucidate how morphological variation interacts with the ecological surroundings [10,11,12]; it looks for adaptive explanations for specific shapes, considering the external environment as the main evolutionary cause of the observed morphology [13]. Understanding how shape interacts with ecological, genetic, biomechanical or other factors is helpful, since traditional ecologists are interested in associating different phenotypes with environmental variation. The ecomorphological approach in ichthyology has had a relevant role in understanding fish diversity [10,14,15], since fish have great phenotypic and ecological diversity, live in a wide variety of ecological niches and have complex reproductive methods and complex sets of foraging, locomotor, respiratory, reproductive and sensory structures that allow them to live in different environments [16,17]. This has provided a wide field for studies of the relation between environment and biological shapes [10,14,18,19,20].

Most of these phenotypic studies have been possible thanks to the change from field descriptions to quantitative science [1], due to advances in technologies and statistics that allow morphological data to have greater complexity, comparing parameters among study groups and controls and establishing relations between them, which makes it possible to explain the patterns found [1,21,22]. Comparative anatomy originally used linear variables such as measurements, distances, angles and proportions, which were analyzed with multivariate statistics and expressed as a set of coefficients and graphs. Sometimes, it was difficult to interpret variations in size and shape, given the high correlations of linear variables with size and, although a method (allometry) was devised to remove the size effect, the disparity of the results in traditional morphology (TM) was not satisfactory. Geometric morphometrics (GM) arose due to the limitations of TM. GM allows analyzing the shape of organisms and/or structures using the geometric space and multivariate statistical methods that have better biological interpretation [23]. GM is based on digitizing X and Y coordinates (and Z, in 3-D morphometrics) of the positions of landmarks [24,25,26]; the generalized Procrustes analysis (GPA) [27] eliminates variations due to scale, rotation and translation. The results are then analyzed with multivariate statistics (e.g., ANOVA, regression) [28,29], which also provide graphic analyses that allow for quantifying and visually understanding the morphometric variation within and among populations [22,30]. Cadrin [31] suggested that morphometric analysis provides a unique perspective for the study of population structure. In recent decades GM has been fundamental in the study of populations, especially in fish; some studies have used morphometric analysis to identify fish stocks [5,32]; Alarcón-Durán [33] studied six isolated populations of silverside (*Chirostoma humboldtianum*), reporting that the habitat and feeding habits influence shape significantly in the different populations. Narváez et al. [34], analyzed the variation in shape of two populations of domestic and naturalized *Oreochromis niloticus*, finding morphological disparity due to adaptations to the habitat.

*Caquetaia kraussii* is a cichlid endemic to Colombia and Venezuela of great commercial importance [35,36]. It lives in a variety of habitats, from lotic areas such as the middle and lower parts of the Sinú, Cauca, Magdalena, San Jorge, Cesar, and Arauca Colombian Rivers, and lentic habitats such as freshwater or low salinity marsh with submerged vegetation [37,38]. Regarding morphological information on the species, the presence of sexual dimorphism between different localities along the “Canal del Dique” (Colombia) has recently been described, which could be associated with environmental variation [39]. Therefore, the objective of this study is to quantify the phenotypic variation in *C. kraussii* using advanced morphological tools that quantify shape in different populations of *C. kraussii* in the “Canal del Dique”, Colombia, and to evaluate associations between different ecological parameters of the study sites and the morphological differentiation of its populations.

## 2. Materials and Methods

**Figure 1:**
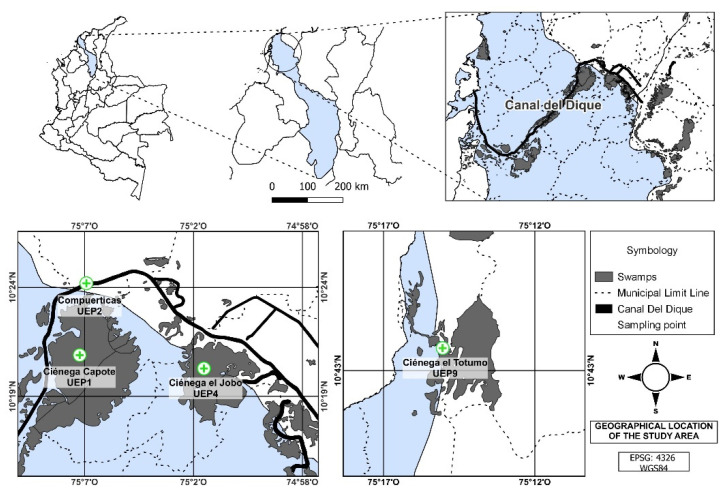
Map of the sampling points. Upper-right: Canal del Dique (sub-regions: UEP1: Capote Marsh, UEP2: Compuerticas UEP4: Jobo Marsh); and lower-left: Canal Norte (UEP9: Totumo Marsh).

The study was carried out in the Bolivar Department, specifically at “Canal del Dique” and “Canal Norte”, including the municipalities of Arjona, Arroyohondo, Calamar, Mahates, San Cristóbal, Santa Catalina, San Estanislao de Kostka, Soplaviento and Villanueva. We established four sampling points (SP), one in Canal Norte (SP9) and three (SP1, SP2 and SP4 in the sub-region of the Canal del Dique (Figure 1); the latter area is a flood plain formed by a complex of wetlands composed of marsh that slow the flow of the trough, which has a length of 113 km from the town of Calamar to its mouth in the Cartagena bay [40,41].

### 2.1. Field Work

We performed bimonthly four-day visits from December 2020 to October 2021. Live individuals of *Caquetaia kraussii* were collected by artisanal fishers with cast nets and trammel nets from the study areas. GPS positions were recorded for the fishing areas and each of the sampling points; temperature, pH and dissolved oxygen were measured with a field multiparameter and a pH meter.

Individual fish were identified in situ using the field guide Fish of the Andes of Colombia and the Catalogue of Continental Fish Resource of Colombia [42,43]. We analyzed 115 individuals of *C. kraussii* from the four areas studied: UEP1-Ciénaga de Capote, with 47 individuals, although little studied, is known to be located within a very important swamp complex for the food security of the surrounding community; likewise, it is known that the Ciénaga de Capote is the largest in the complex and suffers from clogging processes that facilitate the accumulation of sediments, reduction of the depth and increase in water temperature. UEP2-Canal del Dique/Compuerticas, with 37 individuals, is a sampling point on the dike channel and lotic water system. For UEP4-Ciénaga del Jobo/San Cristóbal, with 23 individuals, there currently is not much information about the swamp. It is known that the water mirror has been disappearing and the emerged land has been used for activities such as grazing and agriculture. A part of the surrounding area is maintained as a wetland, in which there are still some patches of intervened secondary natural forests. Finally, Ciénaga del Totumo (UEP9), with eight individuals, is an estuarine ecosystem located in the north of the department of Bolívar with an average surface area of 15 km^2^, an area of 1358 hectares and a perimeter of 31,754 m [44,45].

### 2.2. Geometric Morphometrics

**Figure 2:**
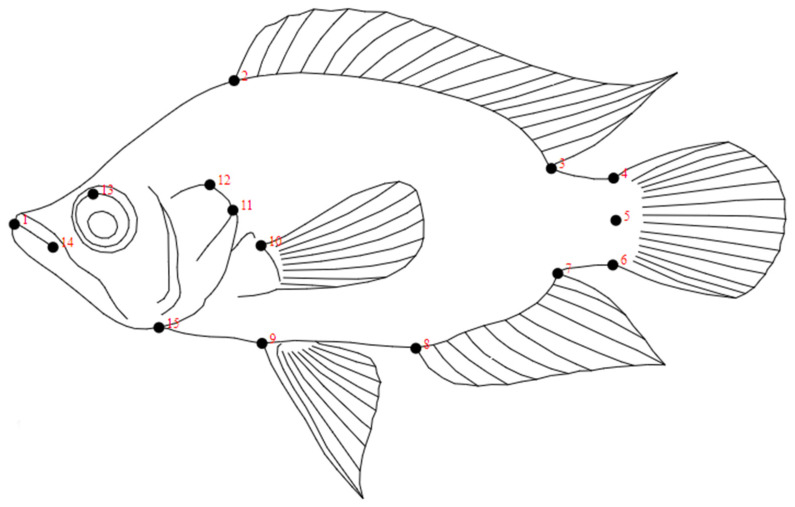
Graphical representation of *C. kraussii* showing the locations of 15 landmarks following Hernandez et al. [39].

Images were obtained by fixing individuals on white Styrofoam with pins in the anterior–posterior position with the fins extended; a ruler was included in the photos to establish the scale. Photographs were taken with a high-resolution FUJIFILM X-T2 24-megapíxel camera; TPS programs was used to transform the photos to TIF format [46]. Fifteen landmarks were digitized using Hernandez et al. [39]’s landmark map for *Caquetaia kraussii*; they are illustrated in Figure 2. The landmarks were digitalized and transformed to two-dimensional coordinates using the software tpsDig2 [47].

The software MorphoJ 1.07a [48] was used to process the Cartesian coordinates in a generalized Procrustes analysis (GPA). This analysis superimposes the resulting configurations of all analyzed individuals, fitting them to the size of centroid 1 and eliminating the rotation and translation of the images [27]. This also allows describing and comparing the shape of the fish, since the Procrustes analysis calculates the mean configuration that summarizes the configurations of all the landmarks [28,49,50].

The measurement error in landmark digitalization was estimated by measuring a sample twice. A Procrustes ANOVA was used to determine if the mean squares of the individuals was less than the error [51]. The morphospace of the geometric shape was examined with a principal components analysis (PCA), generating a scatterplot of the first two dimensions using the covariance matrix of the individuals. Finally, to compare among sites, the average shape of each locality was extracted using the covariance matrix of shape means; the thin-plate-spline were graphed and superimposed. To evaluate if size influences shape (allometry), we used a multivariate regression with shape as the dependent variable and centroid size as the independent variable, which helps to identify if there are allometric effects on shape [52]. A two-block partial least squares analysis [53] was used to determine the influence of environmental parameters on shape [54]; the parameters measured were dissolved oxygen, temperature and the pH of the water. Because it is important to understand the effect of every variable on shape, a PCA was performed on the scaled covariables in order to determine the influence of each variable on the body shape. In order to calculate if there is any significant relationship between the covariates on shape, an ANOVA under a linear model was performed under the first three components, which have the ecological information on the morphological shape. This analysis was performed using the geomorph R package [55,56].

## 3. Results

**Figure 3:**
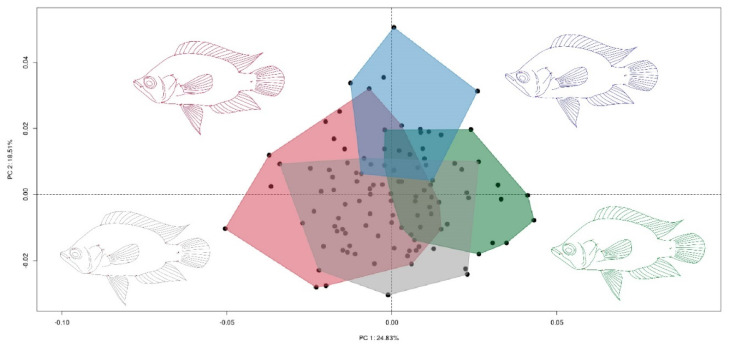
Principal components analysis of *C. kraussii*. Red: UEP1; green: UEP2; grey: UEP4; blue: UEP9.

The errors estimated by the Procrustes ANOVA (MSERROR 0.0000069 < MSINDIVIDUAL 0.0000773) indicated that there was considerably more variation among individuals than measurement error. In the PCA, the first three components explained 53.28% of the shape variation in of *C. kraussii* (PC1: 24.82%; PC2: 18.51%; PC3: 9.94%) (Figure 3).

**Figure 4:**
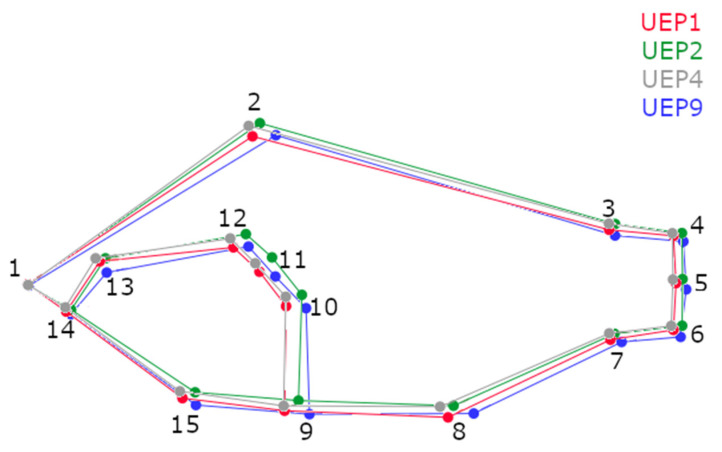
Graphic representation of variation in the mean shape of *C. kraussii* of the populations and their reference points. Red: UEP1; green: UEP2; grey: UEP4; blue: UEP9.

The average shape PCA (Figure 4) showed variations e among the populations, indicating that UEP1 and UEP4 were different than UEP2 and UEP9.

The variations in UEP1 and UEP4 have a conserved and compact shape, but with small variations. UEP1 shows a lowering of points 2 and 8, which represent the first dorsal and anal fins, respectively, with respect to UEP4. UEP2 and UEP9 have differences in shape compared to the other populations, with a more elongated shape; landmarks 4, 5, 6, 10, 11 and 12 (dorsal insertion of caudal fin, ventral insertion of caudal fin, central insertion of caudal fin, insertion of pectoral fin, posterior border of the operculum and dorsal border of the preoperculum, respectively), show longitudinal displacement towards the caudal part of the fish. In UEP9, landmarks 7 and 8 (posterior insertion of anal fin and first anal spine) are lower than in the other three populations. UEP2 have narrower bodies, more flattened ventral area and more erect caudal area than UEP1, UEP4 and UEP9.

**Figure 5:**
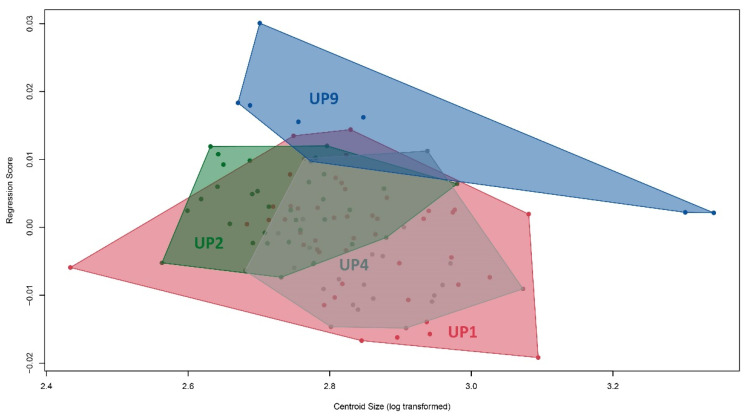
Multivariate regression between body shape and centroid size of *C. kraussii.* Colors represent localities: red: UEP1; green: UEP2; grey: UEP4; blue: UEP9.

The multivariate regression of size on shape (Figure 5) showed that the allometric effect was only 2.73%; individuals of UEP2 were smaller than the other localities and UEP1 and UEP4 were the largest; those of UEP9 were intermediate in size.

**Figure 6:**
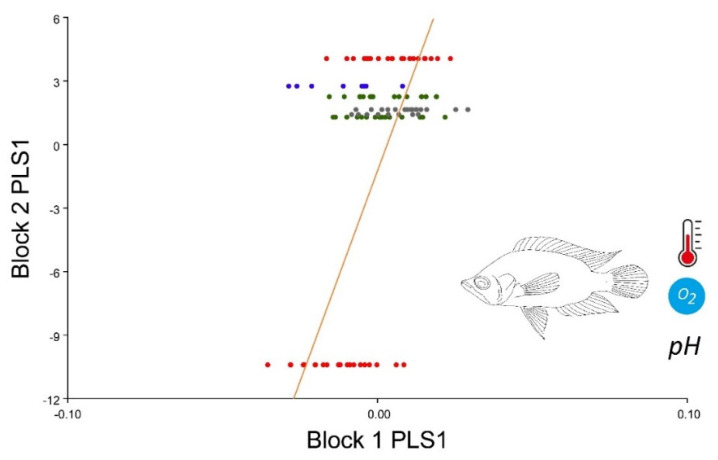
Partial least squares analysis of the body shape of *C. kraussii* and the environmental parameters of temperature, pH and dissolved oxygen (DO). The first horizontal of the graph Block 1 PLS1 contains shape, and the vertical axis Block 2 PLS1 contains the environmental variables. The colors represent localities: red: UEP1; green UEP2; grey UEP4; blue: UEP9.

The graph of PLS associated with the three ecological variables and body shape (Figure 6) shows a positive correlation between environmental effects and shape in the four populations (Figure 6). The PCA of the covariates showed a moderate influence of dissolved oxygen on shape indicated by PC2 (ANOVA: Z: 2.7622 *p* = 0.003), and a contrasting effect of the variables T°C and pH indicated in PC3 (ANOVA: Z: 5.0011 *p* = 0.001). These two variables explained 10% of the shape of *C. kraussii* and are directly correlated with the effect of the currents on the studied populations (greater current > DO). These variables were corroborated by an ANOVA (Table 1), which confirmed the results, showing high significance for the effects indicated in PC2 and PC3.

## 4. Discussion

This study demonstrated morphological differentiation among *C. kraussii* from different localities between the “Canal del Dique” and “Canal Norte”. The results showed that the body shape of UEP1 and UEP4 were similar, as were individuals of UEP2 and UEP9. These results appear to indicate the effect of environmental pressures on shape in the different populations of *C. kraussii*, since both UEP1 and UEP4 live in lentic marsh areas and have a robust and compact body shape and greater average body sizes. In contrast, UEP2 and UE9 inhabit lotic environments in the dike canal and in the entrance canal of the Totumo marsh, respectively, and have more slender and lengthened shape compared those in marsh areas, as well as greater operculum sizes and smaller body sizes. A similar pattern was reported by Perazzo et al. [57] in populations of *Bryoconamericus iheringii*, in which individuals of lentic habitats had larger body sizes and smaller operculum sizes than individuals of lotic environments, suggesting an effect of water velocity on body shape, favoring a more hydrodynamic shape, as well as larger gill opercula to facilitate oxygen exchange. Schofield et al. [58] showed an effect on the behavior of the cichlid *Cichlasoma urophthalmus*, demonstrating that individuals in environments with low oxygenation had a significantly greater frequency of bubble-holding, surface swimming and aggression, concurring that larger body sizes are associated with environments with greater oxygenation. Crispo and Chapman [59] reported a similar shape pattern in the cichlid *Pseudocrenilabrus multicolor*, where individuals in habitats with low oxygenation had more compact shapes than those that inhabit areas with high oxygenation.

The PLS analysis indicated that about 90% of the variation in body shape explained by PC1 was not due to the environmental variables measured. This agrees with the results of Hernández et al., 2022, which show marked sexual dimorphism, suggesting that the body shape of males of *C. kraussii* is more conserved, submitted mostly to sexual selection pressures, while the body shape of females appears to be affected by environmental pressures, from which it may be inferred that most body shape variation is subject to sexual selection [39]. By contrast, PC2 and PC3, which together explained about 10% of the body shape variation, are mostly (≈90%) determined by the dissolved oxygen and the T°-pH combination, respectively. This agrees with several studies that explain how these environmental parameters may influence phenotypic variation in fish [60,61,62]. If dissolved oxygen is a proxy for water movement, we may infer that the shape of individuals of *C. kraussii* in different localities responds to environmental pressures produced by the force and intensity of the current, with a significant difference between fish from marsh areas and lotic environments [63]. This may be observed especially in the similarity of shapes of the individuals of UEP2 and UEP9, since they occur in watersheds separated by about 40Km, which is a strong barrier to the exchange of genes between them, supporting the hypothesis that the body shape of the individuals responds to the environmental pressures of the habitats where they live.

Finally, since *C. kraussii* is a species with commercial value [43], as well as a subsistence resource for the local human communities, this study provides valuable information on the effect of environmental variables on the size and shape of the individuals. It is also important to indicate that the body shape of *C. kraussii* may respond to the interaction of multiple environmental factors that act directly and indirectly on the morphology of individuals. For this reason, the present results contribute to the understanding of the mechanisms of evolution of the ecomorphological characteristics of individuals as a function of different environmental variables that act selectively on the shape of the species. Additionally, this work can serve as a basis for future genetic studies (Microsatellites, AFLP), since it is important to know the status of the populations. It will also encourage studying the sampling sites in this study, since they lack relevant and updated environmental information.

## Figures and Tables

**Table 1:** ANOVA to estimate the effect on shape of the first three PC of ecological variables on shape ** represent highly significant value.

	DF	SS	MS	Rsq	F	Z	Pr (<F)
PC1	1	0.001685	0.001685	0.01283	1.5603	1.2106	0.12
PC2	1	0.003574	0.0035741	0.02722	3.3095	2.7622	0.003 **
PC3	1	0.011577	0.0115768	0.08816	10.7197	5.0011	0.001 **
Residuals	106	0.114475	0.00108	0.87179			
Total	109	0.131311					

## Data Availability

Data will be available by request to the authors.
